# Annual Report and Election by the Managers of the Women’s Homœopathic Hospital of Penna.

**Published:** 1890-02

**Authors:** 


### ANNUAL REPORT AND ELECTION BY THE MANAGERS OF THE WOMEN'S HOMŒOPATHIC HOSPITAL OF PENNSYLVANIA.

Messrs. Editors: I submit herewith a newspaper report (Philadelphia *Press*, January 31st) of the annual meeting of the Women's Homœopathic Hospital. I merely add that, as far as personal investigation can enlighten me, I am convinced this report, in regard to the charges made against the management, is entirely correct and truthful.

Edmund J. Lee.

---

### THE WOMEN'S HOSPITAL.

### SECRETARY'S REPORT AND OTHER BUSINESS AT THE ANNUAL MEETING.

[*The Press*, January 31st, 1890.]

The annual meeting of the Women's Homœopathic Association of Pennsylvania was held yesterday morning, at Early's Hall, 1321 Arch Street. Mrs. Edward Longstreth presided.

The following were elected as the Executive Board for the ensuing year: Mrs. M. W. Coggins, Mrs. E. J. Bartol, Mrs. M. T. Keehmle, Mrs. F. B. Skinner, Mrs. Roswell Weston, Mrs. W. S. Bailey, Mrs. H. Wilson Catherwood, Mrs. J. F. Cummings, Mrs. John Lucas, Mrs. Richard S. Mason, Mrs. S. Rodman Morgan, Mrs. G. W. Myers, Miss K. M. Pleis, Miss A. E. Ramberger, Mrs. S. H. Reese, Mrs. E. R. Sargent, Mrs. R. J.

Stokes, Mrs. Jesse W. Thatcher, Mrs. Alfred Tucker, Mrs. M. L. Jackson, Mrs. Alfred W. Sumner, Mrs. Thomas S. Harper, Mrs. A. W. Paxson, Mrs. H. E. Abbott.

The following were elected as an Advisory Board: Joseph Jeanes, Edward Longstreth, Richard S. Mason, John R. Longstreth, Dundas T. Pratt, Paschal H. Coggins, Ellis D. Williams.

The medical and surgical staff as elected, is as follows: Resident physician, Lucy H. Porter, M. D.; consulting physicians and surgeons, Charles G. Raue, M. D., Malcolm Macfarlan, M. D., Walter M. James, M. D., Edward Fornias, M. D.; attending board medical department, Charles McDowell, M. D., John V. Allen, M. D., G. E. Gramm, M. D., C. S. Schwenk, M. D.; surgical department: Duncan MacFarlan, M. D., Carl V. Vischer, M. D.; gynecological department, Theodore Gramm, M. D.; obstetrical department, Jesse W. Thatcher, M. D., Anna E. Dumont, M. D., William F. Berkenstock, M. D., Eliza J. Remick, M. D.; dispensary, Charles McDowell, M. D., E. J. Remick, M. D., L. H. Porter, M. D., G. E. Gramm, M. D., William F. Berkenstock, M. D., José Congosto, M. D.; eye and surgical clinic, Carl V. Vischer, M. D.; dentist, Alexander P. Long, D. D. S.; special staff, P. P. Wells, M. D., William P. Wesselhœft, M. D., Edward Rushmore, M. D., Alice P. Campbell, M. D., Levi Hoopes, M. D., Eugene D. Nash, M. D.

The following is an extract from the annual report of the Secretary:

"The Managers of the Women's Homœopathic Hospital sincerely regret that one of the hospital dispensary physicians, whose name has been made unpleasantly public, should have been so delayed in complying with a law regarding registration, as to have become amenable to a charge of violation of a wise statute, meant for the protection of the public. This was not her intention, as the testimony given during her trial has fully proven.

"The arrest of this physician on the complaint of one of her own profession was the initial step in what we believe to be a *concerted movement*, whose object seems to be to cripple the usefulness of the hospital. While her trial was pending, the resignation of a number of the staff and the two resident physicians were presented to the Board with no reasons given. After vain endeavors on the part of the president and members of the Board to learn from the resident physicians, who would have been supposed to know the existence of any cause grave enough to demand their withdrawal, and who had been considered faithful enough, to the confidence placed in them, to give timely notice to the Executive Board, of any danger imperilling its interests. The resignations were accepted at the first meeting of the Board held thereafter."

At the same meeting the following preambles and resolutions were unanimously adopted, the Board being polled, and each member present heartily accepting them.

"'WHEREAS, It has recently been publicly asserted or intimated that great diversity exists in this Board as to the treatment to be applied in its hospital, growing out of the widely-differing views of certain of its members, it seems a fitting occasion to briefly state and emphasize the objects and purposes of this Association so far as they relate to the above matter; we therefore declare:

"'*First*, That this is a Homœopathic Hospital;

"'*Second*, That it is controlled by a Board of women managers, which is non-sectarian in its composition;

"'*Third*, That while in such a Board there have naturally and inevitably been differences of opinion relative to matters of hospital management, there has never been any proposition, motion, or suggestion from any faction or individual member of the Board, providing for or tending toward any change from *pure Homœopathy* as the sole and exclusive treatment in its hospital;

"'*Fourth*, As a further expression of the views and intentions of this Board, be it

"'*Resolved*, That while this Board recognizes the undoubted right of each of its members to her individual opinions upon any and all subjects, it would promptly resent and resist any such assertion of opinion as would divert this hospital from the purposes of its creation—the administration of Homœopathy.

"'*Resolved*, That we cordially invite the investigation of our hospital management by the proper officers of the Commonwealth of Pennsylvania at any time or in any manner that may seem best to themselves.'

"This action of the Board was called forth by vague rumors outside the hospital that Christian Science was used as a means of cure in its wards, and had usurped the place of Homœopathy. At a subsequent meeting of the Board a letter was presented from one of the staff physicians, asking definite information concerning the rumors in regard to the use of Christian Science in the hospital. At the same meeting the Board was polled on the question whether Christian Science had been taught or practiced in the hospital, and each member stated plainly that from her own observation she had never known Christian Science or anything of that nature to be taught or practiced in the hospital.

"The following resolution, framed on these statements, was then unanimously adopted by the Executive Board, and sent to the physician in answer to his inquiry:

"We, individually, and as a Board, deny that Christian Science has ever been practiced, taught, or advocated either to patients or nurses in our hospital, and that the charges against us have been equally conspicuous for emphasis of assertion and absence of detail.

"This intrusion upon our regular routine duties has been annoying and needless, but with the re-organized staff of intrusted and faithful physicians, our work is going on more firmly grounded, and with better results that ever. We enter upon another year with renewed strength and courage, better equipped in all material respects than ever before, with a full and efficient staff, who are nobly assisting the management—in carrying forward the work of the hospital on the lines laid down in its charter."

